# Influence of Genetic Background and Tissue Types on Global DNA Methylation Patterns

**DOI:** 10.1371/journal.pone.0009355

**Published:** 2010-02-23

**Authors:** Howard H. Yang, Nan Hu, Chaoyu Wang, Ti Ding, Barbara K. Dunn, Alisa M. Goldstein, Philip R. Taylor, Maxwell P. Lee

**Affiliations:** 1 Laboratory of Population Genetics, Center for Cancer Research, National Cancer Institute, Bethesda, Maryland, United States of America; 2 Genetic Epidemiology Branch, Division of Cancer Epidemiology and Genetics, National Cancer Institute, Bethesda, Maryland, United States of America; 3 Shanxi Cancer Hospital, Taiyuan, Shanxi, People's Republic of China; 4 Basic Prevention Science Research Group, Division of Cancer Prevention, National Cancer Institute, Bethesda, Maryland, United States of America; Cleveland Clinic, United States of America

## Abstract

Recent studies have shown a genetic influence on gene expression variation, chromatin, and DNA methylation. However, the effects of genetic background and tissue types on DNA methylation at the genome-wide level have not been characterized extensively. To study the effect of genetic background and tissue types on global DNA methylation, we performed DNA methylation analysis using the Affymetrix 500K SNP array on tumor, adjacent normal tissue, and blood DNA from 30 patients with esophageal squamous cell carcinoma (ESCC). The use of multiple tissues from 30 individuals allowed us to evaluate variation of DNA methylation states across tissues and individuals. Our results demonstrate that blood and esophageal tissues shared similar DNA methylation patterns within the same individual, suggesting an influence of genetic background on DNA methylation. Furthermore, we showed that tissue types are important contributors of DNA methylation states.

## Introduction

Epigenetic information is contained within DNA and protein components of chromatin; the former is represented mainly by 5-methylcytosine modification of DNA [1], whereas the latter has more complex constituents consisting of histone and non-histone proteins as well as their post-translational modifications [2]. Gene expression patterns are established and maintained by epigenetic information in the chromatin during the development of an organism. Epigenetic plasticity provides the mechanism for tissue differentiation and physiological response to the changing environment; abnormal regulation of epigenetic information is involved in many human diseases including cancer. Epigenetic alterations are hallmarks of human cancer (for reviews see [3], [4]). Global DNA hypomethylation was first observed in human cancer nearly 25 years ago [5], [6]. Subsequently, increased DNA methylation in the promoter region was found to be a common mechanism by which tumor suppressor genes are inactivated in human cancer [7], [8], [9]. The hypermethyation often occurred in CpG islands near the transcription start site (TSS) of the inactivated genes. The annotation of CpG islands for the entire human genome is available in public databases such as the NCBI and UCSC databases. In addition to altered DNA methylation, an array of histone post-translational modifications is often abnormal in human cancer, and proteins responsible for modifying chromatin are similarly altered [10], [11], [12]. Recently, epigenetic investigation has shifted to the genome-wide scale using high throughput technology, and a number of methods have been developed to study DNA methylation at this level. [13], [14], [15], [16], [17].

The DNA methylation state in blood, tumor, and adjacent normal tissue can provide an insight into epigenetic mechanisms that contribute to carcinogenesis [18], [19], [20], [21]. DNA methylation patterns from white blood cells (designated as “blood”) can potentially be used to diagnose cancer early, assess prognosis, and monitor response to chemotherapy and radiation therapy. We previously demonstrated that genetic background influenced global epigenetic states characterized by histone H3 lysine 9/14 acetylation and lysine 4/9/27 methylation [22]. A recent paper reported that DNA sequences determine allele-specific DNA methylation [23]. A longitudinal family-based study indicated that global DNA methylation changed over time and displayed familial clustering [24]. Furthermore, a comparison of MZ (monozygotic) and DZ (dizygotic) twins showed that DNA methylation differences in the buccal cells of DZ twins were higher than those of MZ twins [25], suggesting that heritability may include DNA methylation in addition to DNA sequences.

Esophageal squamous cell carcinoma (ESCC) is one of the most common malignancies in China. Incidence rates of ESCC vary widely in different geographic regions. Shanxi Province in north central China is a region that has a very high esophageal cancer rate. Within high-risk regions, there is a strong tendency toward familial aggregation, suggesting that genetic susceptibility, in conjunction with environmental exposures, plays a role in the etiology of ESCC [26], [27]. Previously, we identified several chromosomal regions of loss of heterozygosity (LOH) and copy number (CN) alterations in ESCC using microsatellite markers and the Affymetrix 10K SNP array [28], [29]. More recently we mapped many genomic regions that showed copy number gain or loss, thus paving the way for identifying oncogenes and tumor suppressor genes in the future [30]. Despite progress in the genomic analysis of ESCC, we know little about global DNA methylation level in normal or tumor cells in esophageal tissue. In this study, we set out to characterize global DNA methylation in three different tissues, blood, normal, and ESCC, from 30 individuals. We found that both genetic background and tissue types impact global DNA methylation.

## Results and Discussion

Our study design had three samples in each set; the samples consisted of DNA from blood, tumor, and adjacent normal tissue from the same individual. We analyzed 90 samples (30 sets, 3 samples in each set). Global DNA methylation was determined using methylation-sensitive Hpa II digestion followed by hybridization to Affymetrix 500K SNP arrays [31]. Because our goal was to assess methylation in a quantitative manner, it was necessary to factor the underlying variation inherent in the DNA among tested individuals into the methylation score. Conventional genotyping without Hpa II digestion on the 90 samples supplied the baseline DNA variation information. To evaluate the quality of genotype experiments, we compared genotype calls that were generated using the Affymetrix Gtype 4.0 software between blood and normal experiments. The genotype call rates generally exceeded 99% for blood and normal esophageal DNA, and the concordances between the genotype calls of the two tissues were in the range of 98.8%–99.8%, demonstrating high quality of the genotype data. For quantitative evaluation of DNA methylation data, we applied a method that we previously developed to analyze chromatin immunoprecipitation (ChIP) data generated using the Affymetrix SNP array experiments [22]. This method is briefly summarized in the Materials and Methods section.

**Figure 1:**
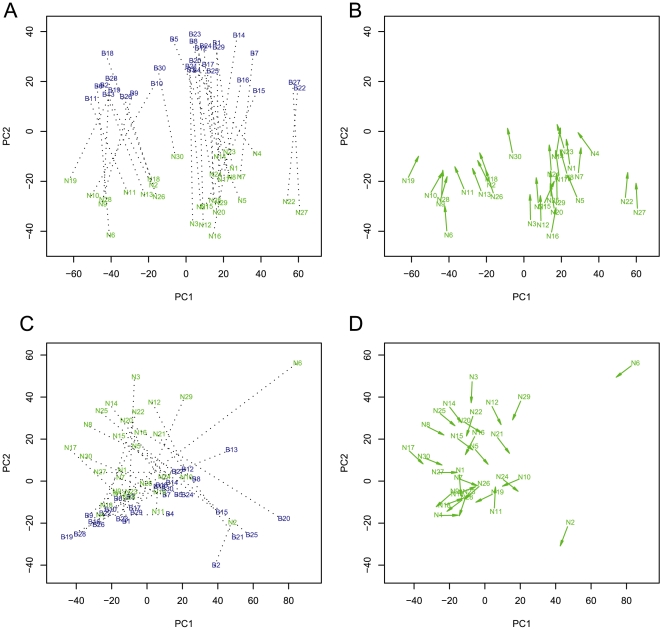
Analysis of DNA methylation patterns in blood and esophageal tissue from 30 ESCC patients. Figure 1A shows the PCA using methylation measurements. Two clusters are evident, corresponding to blood and normal samples. Samples are labeled with blue (patient blood, letter B) and green (patient normal, letter N). The numbers indicate patients. Blood and normal samples from a single patient are connected by a dashed line. The dashed lines are mostly parallel to PC2 axis due to nearly identical PC1 scores between blood and normal esophageal tissue from a single individual. Figure 1B shows unit length direction arrows of the same data as Figure 1A. An arrow emanates from a normal sample and points to blood. The direction of the arrow is identical to that of the dashed line in Figure 1A. Figure 1C shows similar PCA using quantitative DNA measurements. Data processing and analysis is similar to Figure 1A. The DNA measurements serve as a control for variation in the samples. Figure 1D shows the plot of arrows using the data from Figure 1C. It is evident that the arrows in Figure 1D (for DNA) are randomly orientated due to different PC1 scores between the two tissues from the same single individual.

To explore the variation of DNA methylation patterns in relation to tissue types and genetic background, we performed principal components analysis (PCA) to visualize DNA methylation patterns among samples in reduced dimension space (see Materials and Methods for details about PCA analysis). We projected the samples using the first two principal components (PC1 and PC2). Each principal component (PC) is a linear combination of DNA methylation scores measured from SNPs across the whole genome with certain attributes. The Sty I Affymetrix SNP chip contains 238,304 SNPs. Of these SNPs, 62,765 were contained within Affymetrix probes homologous to regions in the sample DNA with the attributes required for our methylation analysis: (1) the Sty I fragment has at least one Hpa II site; (2) the SNP within the fragment does not overlap an Hpa II site; and (3) the SNP is located on an autosome. The results of PCA using data from these 62,765 SNPs are shown in Figure 1A (note: a similar result was observed using the data from the Nsp I chip). In this study PC1 and PC2 provided an efficient way to visualize relationships among the samples in two-dimension space. Two sample clusters were evident, which corresponded to the blood and normal samples, supporting the idea that DNA methylation is dependent on tissue types. Although PC1 and PC2 captured only 24% of the total variation, they grasped the biological variation due to different tissues and different individuals. Analyses involving additional principal components didn't yield any new insight into the nature of DNA methylation.

**Figure 2:**
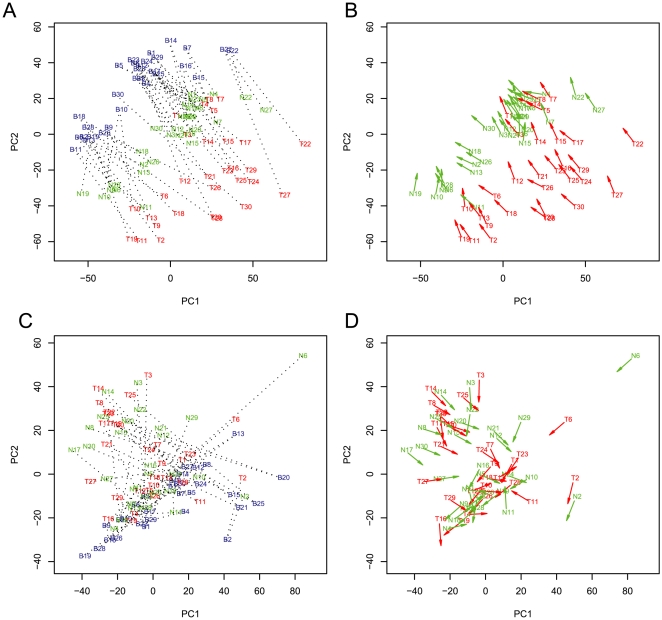
Analysis of DNA methylation patterns in blood, normal esophageal tissue, and tumors. The analysis is similar to Figure 1 except that we include 30 tumors from the same individuals. Figure 2A shows the PCA using methylation measurements. Three clusters are evident, corresponding to blood, normal, and tumor samples. Samples are labeled with blue (patient blood, letter B), green (patient normal, letter N), and red (patient tumor, letter T). Figure 2B shows unit length direction arrows of the same data as Figure 2A. The green arrows emanate from normal and point to blood samples whereas the red arrows start from tumors and point to blood samples. Figure 2C shows similar PCA using quantitative DNA measurements. Data processing and analysis is similar to Figure 2A. The DNA measurements serve as a control for variation in the samples. Figure 2D shows the plot of arrows using the data from Figure 2C. It is evident that the arrows in Figure 2D (for DNA) are randomly orientated due to different PC1 and PC2 scores between different tissues from a single individual.

A unique feature of our study design was the comparison of DNA methylation among multiple tissues from a single individual. Of particular interest was the comparison of DNA methylation in blood versus normal tissue. We wanted to know whether DNA methylation from blood and esophageal tissues from the same individual shared a similar pattern. This central question addresses the potential role of genetics in determining tissue methylation [22], [23], [25], [32]. In the graph shown in Figure 1A, we noted that blood and normal esophageal samples from a single individual had similar scores in PC1 (paired samples are connected by dotted lines in Figure 1A), indicating that these two samples from an individual had a similar DNA methylation level as measured by the PC1 score. To illustrate that the two tissues from the same individual had similar PC1 scores, we generated a plot using a unit length arrow that emanated from the normal sample and pointed to the paired blood sample in the direction corresponding the line shown in Figure 1A for each individual (Figure 1B). These arrows clearly point in the same direction, indicating similar PC1 scores for the paired samples from the same individual. Our data showed a greater similarity in methylation between the two tissues from a single individual, as demonstrated by similar methylation in PC1 score. One interpretation of this result is that genetic background can influence DNA methylation, as we have also previously demonstrated at the level of chromatin modifications [22]. To evaluate whether DNA sequences alone could explain this pattern of PCA, we also performed similar analyses using the measurements of genomic DNA from the genotyping experiments (Sty experiment without Hpa II digestion). PCA was also performed using quantitative values of the hybridization signals [22] rather than the conventional interpretative approach, i.e. using genotyping calls generated according to a calling algorithm. Comparing the results from DNA methylation and genomic DNA, we found two important differences: (i) The clusters in the projection of genomic DNA were not well separated (Figure 1C); and (ii) The arrows in Figure 1D and Figure 2D were more randomly distributed than those in Figure 1B and Figure 2B. In the DNA analysis, blood and normal esophageal samples from a single individual produced very different scores in PC1 (Figure 1C and 1D). Thus, the similarities of PC1 scores in DNA methylation from two different tissues of a single individual are specific. To understand the distribution of the arrows, we generated an angle plot (Figure S1). The angles are more uniform and have smaller standard deviations in the methylation data than the DNA data (Figure S1A and Figure S1B, also see Table S1). We conclude that global DNA methylation is affected by the genetic background. The two clusters of samples correspond to the two tissue types (Figure 1A), indicating that tissue type is an important determinant of DNA methylation.

**Figure 3:**
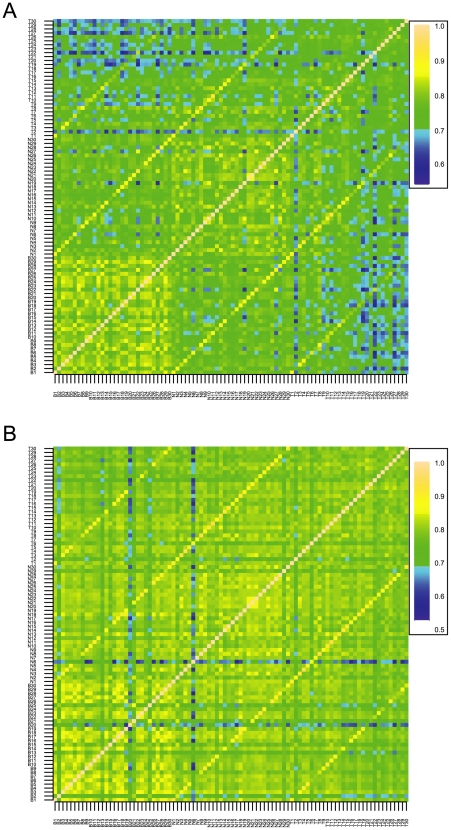
Heat map displays pair wise correlation results for methylation and genomic DNA data. Figure 3A contains methylation data. We have 90 microarray data generated from samples consisting of 3 tissues (blood, normal, and ESCC) from 30 individuals. All pair wise comparisons were analyzed, and Pearson correlation coefficients were plotted in the heat map. Figure 3B has genomic DNA data. Analyses are similar to Figure 3A except for the use of quantitative values from the genotype experiments.

To further characterize the relationship among different individuals and tissues, we performed pair wise correlation analyses and displayed the results of methylation in Figure 3A and DNA in Figure 3B. The results from these analyses support our interpretation of PCA. Specifically, correlations between 3 tissues are apparent in Figure 3B (DNA) revealed by the 3 yellow diagonal lines. The correlations between blood and tumor samples are least because the presence of genomic instability in tumors and some cross-contamination between normal and tumor samples. Correlations in methylation analyses are generally less compared to the DNA analysis. However, the 3 yellow diagonal lines remain, suggesting that DNA methylation is similar among different tissues from the same individual. The main conclusion that genetic background influences methylation is supported by correlation analysis.

We extended the DNA methylation analysis to include tumors from the same individuals. As shown in Figure 2A, three clusters are evident, which correspond to the blood, normal, and tumor samples. PC1 and PC2 capture 23% of the variance in these data. Furthermore, all three tissues from the same individual shared similar DNA methyation angle signature, as demonstrated by a similar direction of the arrows shown in Figure 2B (green arrows point from normal to blood; red arrows point from tumor to blood). As a control, PCA projection using data from DNA analysis showed random distribution of samples (Figure 2C and 2D). Similarly, the angle plots showed narrower distribution and smaller standard deviation from methylation data than DNA data (Figure S1c vs. Figure S1d and Figure S1e vs. Figure S1f, also see Table S1). Thus, global DNA methylation angle signatures in different tissues, including both normal and tumor tissues, are similar in the same individual, indicating a strong influence of genetic background on DNA methylation.

**Figure 4:**
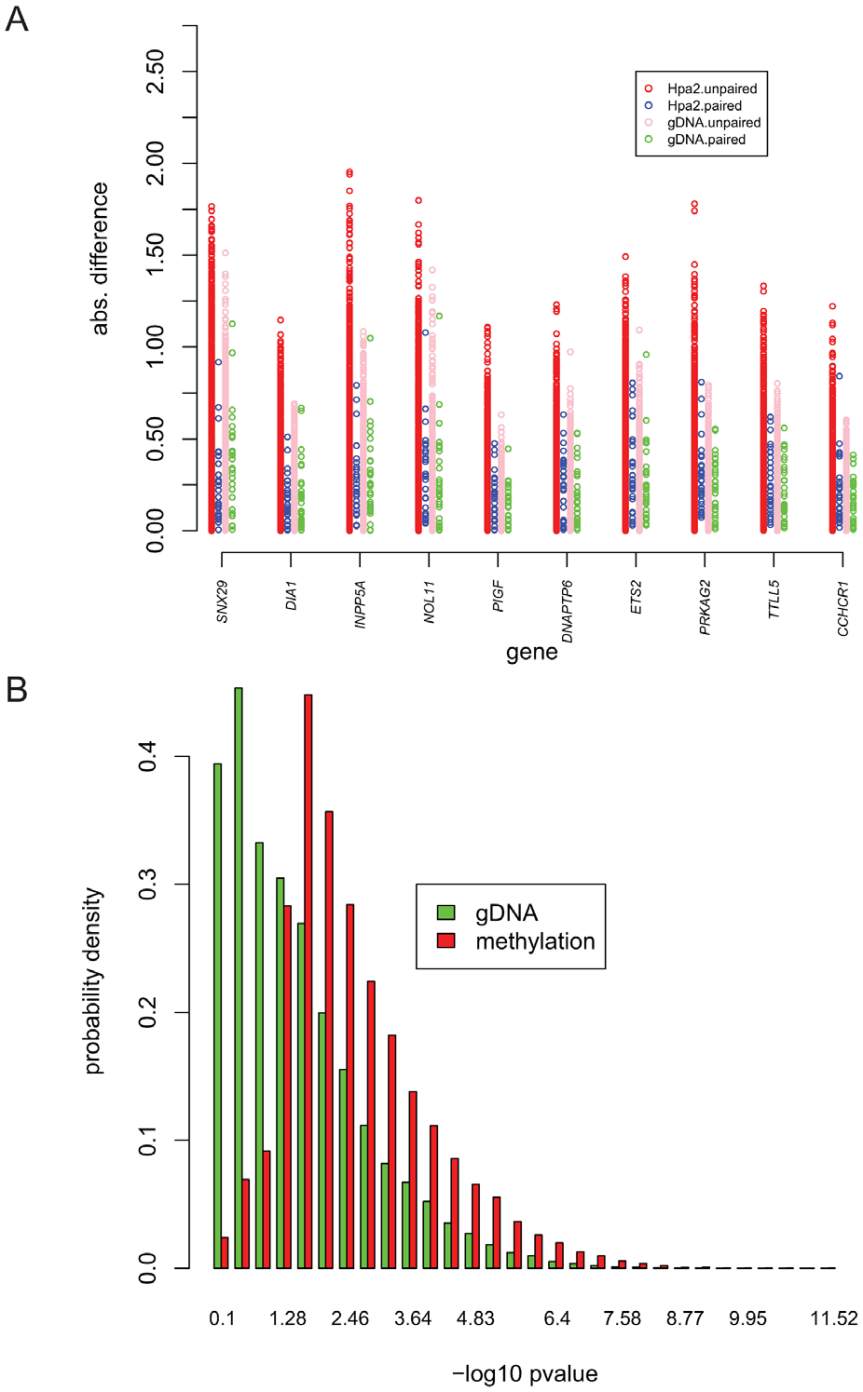
Analyses of DNA methylation difference between blood and normal esophageal tissue for single individuals. Figure 4A displays the genes that exhibit similar methylation measurements between blood and normal esophageal tissue for single individuals relative to the differences between blood and normal tissue for different individuals. Each circle represents a comparison of: (1) methylation measurements between blood and normal tissue (blue from the same individual labeled as Hpa2.paired, 30 data points per gene; red from two different individuals labeled as Hpa2.unpaired, 870 data points) or (2) DNA analysis (green from the same individual labeled as gDNA.paired, 30 data points; pink from two different individuals labeled as gDNA.unpaired, 870 data points). These calculations were carried out for the genes indicated on the x-axis. The SNPs marking the individual genes are rs7203335 (SNX29), rs8190404 (DIA1), rs12780199 (INPP5A), rs3760220 (DKFZP586L0724), rs3768723 (PIGF), rs7605146 (DNAPTP6), rs11254 (ETS2), rs6464151 (PRKAG2), rs2302592 (TTLL5), and rs1265074 (CCHCR1). Figure 4B shows the histogram of p-values in the Ansari-Bradley two-sample test for methylation (red bars, labeled with Hpa2) and DNA (green bars, labeled with gDNA) measurements. For each SNP, the ratio of scales was tested for two samples: one is the methylation differences between blood and normal tissue from 30 pairs of individuals and another is the methylation differences between the two tissues from two different individuals from the 870 pairs resulting from selecting 2 out 30 individuals in all combinations. As a control, we performed similar analyses using data from DNA array experiments. The histogram summarizes the distribution of the negative log10pvalues from the analyses of the methylation and DNA data.

To further analyze genetic influence on DNA methylation, the differences between blood and normal tissue for the same individual and different individuals are shown in Figure 4A for ten selected SNP-marked fragments within the indicated genes. Each circle represents the difference in methylation measurements between blood and normal tissue (blue, from the same individual, labeled as Hpa2.paired; red, from two different individuals, labeled as Hpa2.unpaired) or the difference in DNA analysis (green, from the same individual labeled as gDNA.paired; pink, from two different individuals, labeled as gDNA.unpaired). The absolute difference in methylation is smaller in the two tissues from the same individual in these graphs (blue circles, labeled as Hpa2.paired) than in the two tissues from two different individuals (red circles, labeled as Hpa2.unpaired). To understand the overall distribution of the quantitative effect of genetic influence on DNA methylation across SNPs, we applied the Ansari-Bradley two-sample test, a non-parametric method, to compare the scale parameters for two sets of differences between blood and normal assays: the first set contains 30 differences with one for each individual; the second set contains 870 differences with one for each pair of two different individuals. The method tests the ratio of the scales for the two sets of the differences. The alternative hypothesis is that a ratio less than one means that the scale for the paired differences is less than the scale for unpaired differences. As a control, we performed similar analyses using data from DNA measurements. The ratio of the scales is significantly less than one for many of the tested SNPs (Figure 4B, red bars, labeled as Hpa2, indicate methylation data), showing greater similarity of methylation in two different tissues from the same individual. Some SNPs also showed smaller p-values for DNA measurements (Figure 4B, green bars, labeled as gDNA), reflecting DNA differences in the genetic background of different individuals. Nevertheless, the distribution is clearly shifted to the right (smaller p-value, indicating a smaller ratio of the scales) for the methylation data (Figure 4B, red bars). A non-parametric method was used instead of an F-test because the Shapiro-Wilk normality test showed that the differences were not normal for 65% of SNPs in the methylation data and 70% of SNPs in the genomic DNA data.

In conclusion, we found that DNA methylation characteristics in normal esophageal tissue and blood from the same individual were remarkably similar. Our results indicate that genetic background as well as tissue environment can influence global DNA methyation patterns. This conclusion is consistent with previous studies that showed genetic background affected global chromatin modifications [22], [23], [24], [25], [32].

## Materials and Methods

### Patient Selection

The study was approved by the Institutional Review Boards of the Shanxi Cancer Hospital and the U.S National Cancer Institute (NCI). Written informed consents were obtained for all participants of this study. Patients diagnosed with ESCC between 1998 and 2001 in the Shanxi Cancer Hospital in Taiyuan, Shanxi Province, People's Republic of China, and considered candidates for curative surgical resection were identified and recruited to participate in this study. None of the patients had prior therapy and Shanxi was the ancestral home for all.

### DNA Isolation

Venous blood (10 ml) was taken from each patient prior to surgery and germ-line DNA from whole blood was extracted and purified using the standard phenol/chloroform method. Tumor and adjacent normal tissues were dissected at the time of surgery and stored in liquid nitrogen until use. One 5-micron section was H&E stained and reviewed by a pathologist from NCI as the guide for micro-dissection. Five to ten consecutive 8-micron sections were cut from fresh frozen tumor tissues. Tumor cells were manually micro-dissected under light microscope visualization. DNA was extracted from micro-dissected tumor as previously described [29] using the protocol from Puregene DNA Purification Tissue Kit (Gentra Systems, Inc., Minneapolis, MN).

### Microarray Experiment

We performed genotyping and methylation experiments using the Affymetrix Mapping 500K array set (Nsp I array and Sty I array). The detailed protocol for genotyping can be found at http://www.affymetrix.com/support/downloads/manuals/500k_assay_manual.pdf. Methylation experiments were carried out essentially in the same manner as conventional genotyping assays except for pre-digestion of genomic DNA with Hpa II restriction as described [31]. http://www.affymetrix.com/support/technical/manual/expression_manual.affx

The Gene Expression Omnibus (GEO) accession number for these array data is GSE20123.

### Data Analysis

All statistical analyses were developed using R packages. The analytic method to extract quantitative values from methylation and genotyping experiments was essentially identical to our previous published work [22]. Briefly, the probe level measurements were generated using Affymetrix Gtype software. Microarray data were normalized using a modified RMA method [22]. We used the probe level measurements from standard genotyping experiments that contained 144 case-control blood DNA samples as a reference set to normalize the data. After the normalization process, we obtained measurements at the probeset level for all microarray data, for both DNA and methylation. The correlation data used in the analyses described in this manuscript are included in Table S2 and Table S3.

We used principal component analysis (PCA) to analyze variation among the samples for DNA methylation and genotype data. The PCA method transforms the raw data into a more interpretable form. The raw data are generated as a matrix, in which each column represents one sample and each row, one probeset. The probeset is specific for methylation or genomic signal, depending on the laboratory assay. The PCA method works by rotating this system of matrix coordinates (i.e. probesets or SNPs) in such way that a new set of variables are generated; these new variables are called the “principal components”. A principal component is actually a linear combination of the original variables, each of which is weighted by a different coefficient. The weight represented in each coefficient reflects the degree of contribution that the corresponding SNP makes to the principal component. The resulting principal components are ordered, i.e. numbered, so that PC1 accounts for the largest variance in the data and is followed by PC2, PC3, etc. PC1 and PC2 are the only principal components that are displayed in our 2-dimensional analysis. A major value of the PCA method is reduction in the number of dimensions inherent in the huge amount of microarray data. This allows visualization of the data in two dimensions.

To analyze methylation differences between blood and normal, we compared paired (2 tissues from a single individual) versus unpaired methylation measurements (2 tissues from different individuals). For each SNP, the Ansari-Bradley two-sample test was applied to the comparison in the scale of 30 paired differences and the scale of 870 unpaired differences. As a control, the same test was conducted for DNA measurements. To compare the p-values from the Ansari-Bradley tests for methylation with those for DNA, we included only those SNPs for which paired scale was significantly less than the unpaired scale at the p-value level of 0.05 for either methylation or genomic DNA or both.

## Supporting Information

Figure S1 Angle plot of blood, normal, and tumor samples based on methylation measurements by principal component analysis (PCA). Plots are labeled with green (normal) and red (tumor). The angle plots in Figures S1a and S1b were generated from PCA projects in Figures 1b and 1d. The angle plots in Figures S1c and S1d were generated from PCA projects in Figures 2b and 2d, and contain the arrows from normal to blood. The angle plots in Figures S1e and S1f were also generated from PCA projects in Figures 2b and 2d, but contain the arrows from tumor to blood.
(0.01 MB PDF)

Table S1 Statistical analysis of data in Figure S1. B, N and T are abbreviations for blood, normal and tumor samples. B-N-T represents all samples. The arrows in Figure S1 are defined in the first column of the table. The variances of the angles in the methylation data are significantly smaller than those in the DNA data based on the F-test.
(0.02 MB XLS)

Table S2 Correlation coefficients for methylation experiments. Data are described in Results and Discussion as well as Materials and Methods.
(0.16 MB XLS)

Table S3 Correlation coefficients for DNA experiments. Data are described in Results and Discussion as well as Materials and Methods.
(0.16 MB XLS)
