# Supplementary figures and images for: Influence of Genetic Background and Tissue Types on Global DNA Methylation Patterns
Source: PLoS One. 2010 Feb 23;5(2):e9355. doi: 10.1371/journal.pone.0009355 (PMC2826396; doi:10.1371/journal.pone.0009355)

Methylation

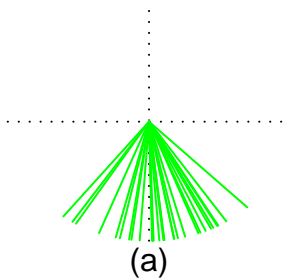

gDNA

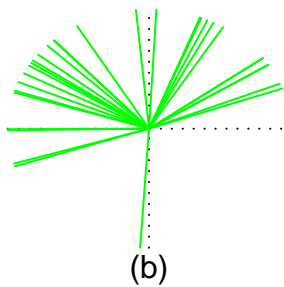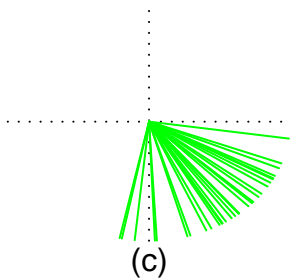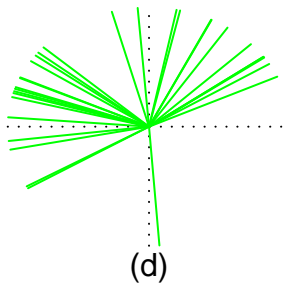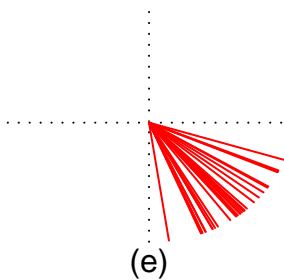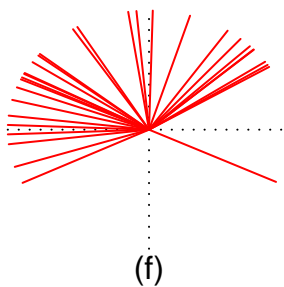

Supplement: Figure S1 — Angle plot of blood, normal, and tumor samples based on methylation measurements by principal component analysis (PCA). Plots are labeled with green (normal) and red (tumor). The angle plots in Figures S1a and S1b were generated from PCA projects in Figures 1b and 1d. The angle plots in Figures S1c and S1d were generated from PCA projects in Figures 2b and 2d, and contain the arrows from normal to blood. The angle plots in Figures S1e and S1f were also generated from PCA projects in Figures 2b and 2d, but contain the arrows from tumor to blood. (0.01 MB PDF) [file pone.0009355.s001.pdf]
